# Kinetic Modeling of Convective and Microwave Drying of Potato Peels and Their Effects on Antioxidant Content and Capacity

**DOI:** 10.3390/antiox12030638

**Published:** 2023-03-03

**Authors:** Fatiha Brahmi, Inmaculada Mateos-Aparicio, Khokha Mouhoubi, Sara Guemouni, Tassadit Sahki, Farid Dahmoune, Ferroudja Belmehdi, Chafiaa Bessai, Khodir Madani, Lila Boulekbache-Makhlouf

**Affiliations:** 1 Laboratory of Biomathematics, Biochemistry, Biophysics and Scientometry, Faculty of Natural and Life Sciences, University of Bejaia, Bejaia 06000, Algeria; 2 Department of Nutrition and Food Science, Universidad Complutense de Madrid, 28040 Madrid, Spain; 3 Agri-Food Technologies Research Center, Targua Ouzemmour Rouad, Bejaia 06000, Algeria; 4 Laboratory of Biomathematics, Biochemistry, Biophysics and Scientometry, Faculty of Natural and Life and Earth Sciences Sciences, University of Bouira, Bouira 10000, Algeria

**Keywords:** potato peels, drying kinetics, forced convection drying, microwave drying, antioxidants, antioxidant capacity

## Abstract

This study deals with drying properties and focuses on the drying kinetics of potato peels (PP) by two processes, namely convection drying (CD) at various temperatures (40, 60, 80, 100, and 120 °C) and microwave drying (MD) at different powers (200, 400, 600, and 800 W). In addition, the effectiveness of the adopted processes was evaluated in terms of antioxidant contents and antioxidant capacity. A total of 22 mathematical models were undertaken to predict the drying kinetics, and the best model was selected based on the highest R^2^ values and the lowest χ^2^ and RMSE values. The Sledz model was the more appropriate for both methods with values of 0.9995 ≤ R^2^ ≤ 0.9999, χ^2^ = 0.0000, and 0.0054 ≤ RMSE ≤ 0.0030 for CD, and the results of MD were 0.9829 ≤ R^2^ ≤ 0.9997, 0.0000 ≤ χ^2^ ≤ 0.0010, and 0.0304 ≤ RMSE ≤ 0.0053. The best drying rates (DR) of PP were assigned to a temperature of 120 °C and a power of 600 W with values of 0.05 and 0.20 kg water/kg dw min, respectively. A potential explanation is that as PP’s moisture content decreased during the drying process, there was a drop in absorption, which led to a reduction in the DR. The energy consumption of both processes was assessed, and it rose with increasing temperature or power. The microwave process reduced the drying time, consumed lower energy, and presented a higher drying efficiency at a moderate power level compared to the convection process. Furthermore, MD preserved antioxidants better compared to CD and improved the antioxidant capacity. Therefore, the proposed microwave process for drying PP is suggested for its expected use in various fields, including the food processing industries.

## 1. Introduction

Potato (*Solanum tuberosum* L.) from the Solanaceae family is commonly recognized as “the king of vegetables” due to the fact that it grows in more than 100 countries [1,2]. According to the Food and Agriculture Organization (FAO), the annual production of potato worldwide was over 359 million tons in 2020 [3]. It has been long used in the diet of Algerians, and according to recent reports from the Algerian Ministry of Agriculture and Rural Development, potato production is around 5.51156 million tons annually in an area of 150,000 hectares. Thus, potato has become the second crop in terms of area, after cereals, in Algeria [4].

Currently, potato utilization designs vary from fresh to transformed preparations, such as potato chips [5]. Potato peel (PP) waste as a by-product is the main waste from the potato transformation agri-food industries. Indeed, about 70 to 140 thousand tons of peels are generated annually across the world [6]. This has an ecological negative impact linked with the waste produced by such industrialized procedures [1].

PP is an inexpensive material for the extraction and production of valuable products, such as dietary fibre, biopolymers, and natural food additives [6]. Many studies are exploring the possibility of the transformation of by-products into ingredients that can be incorporated into various food, cosmetic, or pharmaceutical products. PP can also be a prospective supply of natural antioxidants, such as phenolic compounds [7,8].

Drying is reputed as a crucial factor for the successful preservation and merchantability of raw materials, and its procedures constitute substantial phases in the food and chemical transformation industries [9]. Dehydration of by-products consists of removing water up to a defined amount, usually about less than 15%, to avoid microbial deterioration and to reduce the degeneration caused by biochemical reactions [10]. However, drying procedures can generate modifications that impact on by-product quality. Besides, the dehydration of such natural ingredients is frequently combined with a depletion of bioactive substances, a reduction in antioxidant capacity, and other disadvantageous characteristics [11].

Many drying procedures are employed to preserve natural products, such as oven-, sun, freeze-, and air-drying procedures. In recent years, microwave drying has attracted attention as a substitute drying technique in the agri-food industries. It is fast, more homogeneous, and more power effective compared to conventional methods [10,12,13,14,15].

A substantial number of drying techniques for agri-food by-products are published in the literature. Their basic discrepancies consist of the total drying time, the energy efficiency, and the physical space capacities, among others. An investigation of these variables is commonly needed for the selection of the most economically practical drying process [16].

To the best of the authors’ knowledge, the number of investigations dedicated to the drying of PP is limited and studies that concentrated on the modeling of the kinetics of microwave drying of PP are not available. Nguyen et al. [17] investigated only the air-drying behavior of potato peel and determined and established sorption isotherms. In the same way, Hossain et al. [18] studied the impact of drying processes by focusing only on the steroidal alkaloid content of PP. Additionally, Akter et al. [19] focused on the comparison of the effects of drying cabinet and sun-drying techniques on the physicochemical, antioxidant, and functional properties of dried potato peel flour.

In Algeria, considerable amounts of potato by-products are generated, but to date, the studies that were carried out mainly focused on their use as flocculants for wastewater treatment [20,21] and for the removal of reactive blue 72 from aqueous solutions [22]. They are also used for the production of second-generation bioethanol [23]. More recently, powder and small pieces of dried potato peels were used to fortify yogurt and enhance its antioxidant activity [7]. However, there is a deficiency of data concerning the impact of drying process on antioxidant contents and their capacities. This study represents probably the first methodological investigation of the impact of drying techniques on potato peels. The objective was to research the impact of different temperatures of oven drying and powers of microwave drying on the dehydration of PP by-product to develop a drying protocol for producing PP-based products with a high-added value based on their antioxidant capacity.

The points to be emphasized are (i) the illustrations of drying kinetics by showing the variability of moisture level and drying rate (DR) depending on time and moisture content, respectively; (ii) the presentation of drying kinetic modeling; (iii) the determination of effective moisture diffusivity (D_eff_); and (iv) the calculation of specific energy consumption (SECe) and energy efficiency (EE). All aforementioned parameters were adopted for the two drying methods (connective drying (CD) and microwave drying (MD)), and a comparison was carried out concurrently.

## 2. Materials and Methods

### 2.1. Samples and Reagents

The potato (*Solanum tuberosum* L. Spunta variety) used in this present study was purchased from a local market of Bejaia (Algeria). It was cleaned well with water and rinsed with distilled water to remove all impurities, and then it was wiped dry and peeled using a peeler. The peels were then collected and cut into small square pieces of 0.5 cm^2^.

According to our earlier study, the initial moisture content of PP (87%) was assessed before the drying trials.

Using a caliper, their thickness was also examined, and the average was discovered to be 0.5 ± 0.01 mm.

Ethanol (Biochem Chemopharma, Cosne-Cours-sur-Loire, France) was used for extraction; Folin–Ciocalteu’s phenol reagent (Prolabo, 2 N, Linarsdel Valleıs, Spain), gallic acid (Sigma-Aldrich, 98%, St. Louis, MI, USA), and anhydrous sodium carbonate (Sigma-Aldrich, 99%) were used for total phenolic analysis; aluminium chloride (Biochem Chemopharma, Cosne-Cours-sur-Loire, France, 99%) and quercetin (Sigma-Aldrich, 99%, St. Louis, MI, USA) were used for total flavonoids analysis; 2,2-diphenyl-1-picrylhydrazyl (Sigma-Aldrich, 90%, St. Louis, MI, USA) was used for DPPH assay; sulfuric acid (Biochem Chemopharma, 96%, Cosne-Cours-sur-Loire, France), sodium phosphate (Biochem Chemopharma, Cosne-Cours-sur-Loire, France), and ammonium molybdate (Prolabo, 99%, Linarsdel Valleıs, Spain) were used for phosphomolybdenum assay. Absorbance was measured with a SRECTROSCAN UV–vis spectrophotometer (UK).

### 2.2. Drying Kinetics

Samples of 10 g of PP by-product were homogeneously distributed in a monolayer and were either dried with convection oven drying (CD), where the samples were placed in the middle of the oven applicators (Memmert Model UFB 400, with air circulation, GmbH + Co. KG, Schwabach, Germany) at different temperatures (40, 60, 80, 100, and 120 °C), or with microwave drying (MD), where each sample was placed on the rotating glass of the microwave oven and then placed in the center of the microwave applicators (Maxipower, Model MASMO23S, Beijing, China) at different powers (200, 400, 600, and 800 W).

For the two types of drying, mass was monitored periodically (every 10 min for CD and every 30 s for MD) with an external analytical accuracy of 0.01 g (RADWAG, WPS 600/C/2, Radom, Poland) until it reached a constant weight.

After drying, the samples were ground using an IKA model A11 basic electric grinder (Staufen, Germany). The obtained powders were stored in glass bottles, hermetically sealed, and protected from light and moisture for later analyses.

The mathematical modeling was accomplished, and 22 drying models were identified (Table 1). The obtained equations are related to the moisture ratio (MR) as a function of time as well as the drying rate (DR), effective moisture diffusivity (D_eff_), specific energy consumption (SECe), and energy efficiency (EE), and they were presented in our previous report [10]. The formulas used to calculate the determination coefficient (R^2^), chi-square (χ^2^), and root mean square (RMSE) were also reported by the same authors.

Briefly, the equations employed to calculate the various parameters are as follows:

(1) $$\mathrm{Moisture}\;\mathrm{ratio}:\;MR=\frac{M_{t}-M_{e}}{M_{0}-M_{e}}$$

where *M_t_*, *M_o_*, and *M_e_* designate the moisture content at any point in the drying process, the initial moisture content, and the equilibrium moisture content (kg water/kg dry matter), respectively. *M_e_* values are relatively low compared to *M_t_* or *M_o_,* so the moisture ratio and drying rate are calculated as follows:

*Moisture ratio*: *MR* = *M_t_*/*M*_0_ (2)

(3) $$\mathrm{Drying}\;\mathrm{rate}:\;DR=\frac{M_{t+dt}-M_{t}}{dt}\;$$

where *M_t+dt_* is the moisture content at *t* + Δ*t* (kg H_2_O/kg dry matter), *M_t_* is the moisture content at *t* (kg H_2_O/kg dry matter), and *dt* is the difference in drying time (min).

(4) $$\mathrm{Effective}\;\mathrm{moisture}\;\mathrm{diffusivity}:\;D_{eff}=\frac{F_{0}}{\left(\frac{t}{4L^{2}}\right)}=\frac{-0.101\ln(MR)-0.0213}{\left(\frac{t}{4L^{2}}\right)}$$

(5) $$\mathrm{Specific}\;\mathrm{electric}\;\mathrm{energy}\;\mathrm{consumption}\;(\mathrm{MJ}/\mathrm{kg}\;\mathrm{of}\;\mathrm{water}):\;\;SEC_{e}=\frac{3600E}{M_{s}(X_{i}-X_{f})}\;$$

where *E* is the total electric energy consumption (kWh); *X_i_* and *X_f_* are the initial and final moisture contents (d.b), respectively; and *M_s_* is the mass of dry solid matter (kg).

(6) $$\mathrm{Electrical}\;\mathrm{energy}\;(\%):\;EE=M_{s}(X_{i}-X_{f})\frac{\Delta h_{v}}{3600E}\times 100$$

where Δ*h_v_* is the enthalpy of the evaporation of water (2257 kJ/kg, at 100 °C).

(7) $$\mathrm{Chi}-\mathrm{square}:\;\chi^{2}=\frac{\sum_{i=1}^{N}{\left(MR_{pre,i}-MR_{\exp,i}\right)}^{2}}{N-z}$$

(8) $$\mathrm{Root}\;\mathrm{mean}\;\mathrm{square}:\;RMSE=\sqrt{\frac{\sum_{i=1}^{N}{\left(MR_{pre,i}-MR_{\exp,i}\right)}^{2}}{N}}\;$$

where *MR_exp,i_* and *MR_pre,i_* are the experimental moisture ratio and predicted moisture ratio, respectively, and *N* is the number of observations and is also the number of parameters.

### 2.3. Extract Preparation

Microwave-assisted extraction was used as follows: a volume of 28 mL of ethanol 50% (*v*/*v*) was added to 1 g of powder, and the solution was dried either at different temperatures in the oven or at different powers in the microwave in a flat-bottomed flask. It was placed in a microwave at 400 W for 123 s. After cooling, the solution was filtered using a vacuum pump, and the residue was re-extracted using 15 mL of ethanol under the same conditions. The two fractions collected after filtration were combined and adjusted to 43 mL [25], and the extracts were then analyzed.

### 2.4. Estimation of Total Phenolic Content

For the determination of total phenolic content (TPC), the extracts were submitted to the Folin–Ciocalteu assay. A 2.5 mL sample of water-diluted Folin–Ciocalteu reagent (1/10) was added to the different extracts. The mixture was incubated for 2 min at room temperature, and 2 mL of sodium carbonate (75 g/L) was added; the mixture was then incubated for 15 min at 50 °C and finally cooled in a water-ice bath. The absorbance at 760 nm was immediately measured [26]. TPC was expressed as mg equivalents of gallic acid per 100 g of dry weight (mg GAE/100 g dw).

For the estimation of total polyphenol index (I_280_), the extracts were diluted with water in a 1/30 ratio (*v*/*v*), the absorbance was directly measured at 280 nm, and the value of I_280_ was calculated [25].

The aluminium chloride colorimetric method was adopted to assess total flavonoid content (TFC). Briefly, 1.5 mL of each extract stock solution and 1.5 mL of aluminium chloride were added and mixed well. The absorbance was measured at 430 nm, and the results were expressed as mg equivalents of quercetin per 100 g of dry weight (mg GAE/100 g dw) [7].

### 2.5. Estimation of Chlorophylls a, b and Total Carotenoids

The extraction of chlorophylls and carotenoids was carried out as follows: 0.5 g of dried powder of each sample was separately homogenized with 10 mL of an acetone–water mixture (4:1) for 2 min to form a uniform mass. The homogenates were centrifuged at 5000 rpm (Sigma 2–16 P-Centrifuge, Tuttlingen, Germany) for 10 min. The amounts of chlorophylls a, b, and total carotenoids were determined by measuring the absorbance (UV-Vis Spectrophotometer, spectro scan 50 Shimadzu, Kyoto, Japan) of the supernatants at 663.6, 646.6, and 470.0 nm, respectively [27].

The contents of Chlorophyll a, Chlorophyll b, and total carotenoids were calculated using the following equations:

Chlorophyll a (µg/mL) = 12.25A_663.6_ − 2.25A_646.6_ (9)

Chlorophyll b (µg/mL) = 20.31A_646.6_ − 4.91A_663.6_ (10)

(11) $$\mathrm{Total}\;\mathrm{carotenoids}=\frac{1000\;A470-2.27\;\left(Chla\right)-81.4\left(Chl\;b\right)}{227}$$

### 2.6. Estimation of Antioxidant Activity 

In this study, free radical scavenging activity of the extracts was determined by in vitro assay models, such as DPPH free radical and total antioxidant capacity (phosphomolybdenum assay).

DPPH radical scavenging activity was measured using the method reported by Mateos-Aparicio et al. [28]. Briefly, 2 mL of the reaction mixture containing 150 µL of DPPH (100 μM in methanol), 1000 µL of ethanol, 600 µL of the test solution, and various concentrations of the extracts were incubated at room temperature for 60 min, and the absorbance of the resulting solution was measured at 517 nm. The percentage inhibition of DPPH^•^ radical was calculated by comparing the results of the test with those of the control using the following equation:

Percentage inhibition = (1 − absorbance of test/absorbance of control) × 100 (12)

In the phosphomolybdenum assay, as reported in our previous study [7], the extracts (100 µL) were mixed with 1 mL of the phosphomolybdenum reagent (600 mM of sulfuric acid, 28 mM of sodium phosphate, and 4 mM of ammonium molybdate). Then, the mixture was incubated at 95 °C during 90 min and cooled to room temperature. Subsequently the absorbance was measured at 695 nm. 

### 2.7. Statistical Analysis

In the modeling of drying kinetics, the analysis was performed using the STATISTICA software 8.0 (StatSoft Inc., Tulsa, OK, USA). The drying tests were carried out in triplicate for each temperature and each power. The values are presented as the means of triplicate in all the assays. The average of antioxidant contents and antioxidant capacities of the extracts obtained by the two drying methods at different temperatures and powers were statistically investigated using one-way analysis of variance (ANOVA) with least significant difference (LSD) by STATISTICA. A statistical probability (*p* value) less than 0.05 indicated a statistically significant difference between groups. 

## 3. Results and Discussion

### 3.1. Drying Kinetics

#### 3.1.1. Moisture Content vs. Drying Time

**Figure 1:**
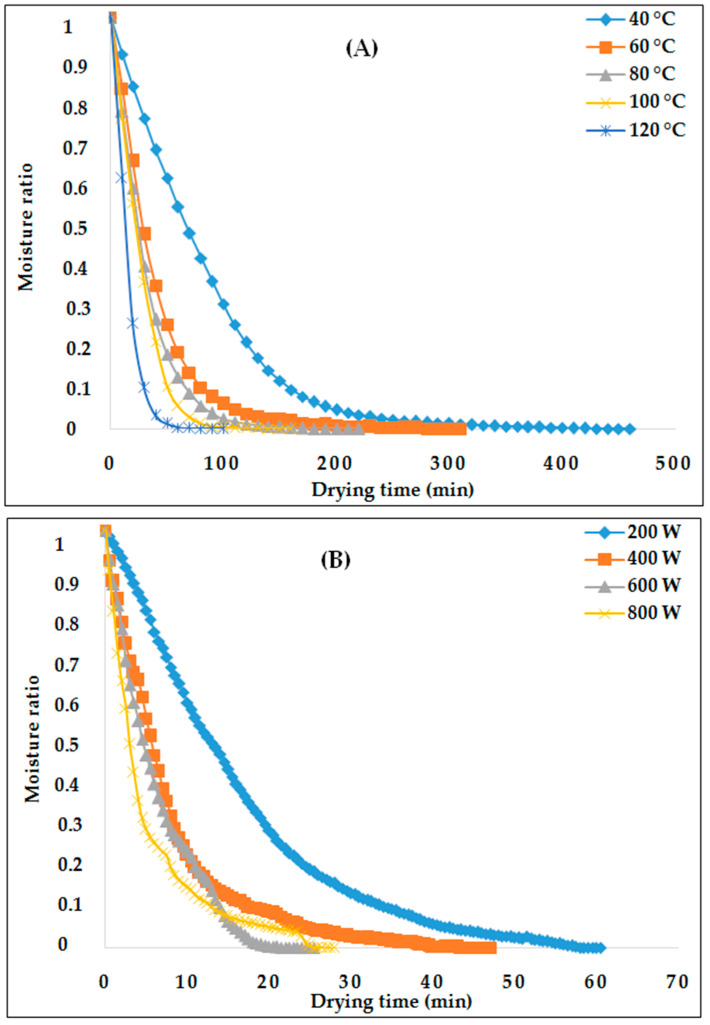
Moisture ratio graphs of potato peels based on (**A**) convective drying and (**B**) microwave drying. The various temperatures adopted in the convection drying (CD) are 40, 60, 80, 100, and 120 °C, and the powers used in the microwave drying (MD) are 200, 400, 600, and 800 W.

The impacts of various temperatures and powers on the moisture content regarding drying time are illustrated in Figure 1A,B. The generated curves resemble those of several other natural by-products. The moisture content is reduced substantially with extended time of drying, and the samples dried at higher temperatures show shorter drying times. 

Aral and Beşe [29] have indicated that a rise in temperature during CD improves the steam pressure in the product, which induces the internal moisture to be eliminated more quickly compared to the surface moisture. Meanwhile, CD is a long procedure and drying happens mainly by moisture diffusion [30]. The same results were observed by Ramos et al. [31] for uvaia (*Eugenia pyriformis* Cambess—Myrtaceae) by-product drying, in which the time ranged from 330 to 1110 min at temperatures from 80 to 40 °C. In the CD of some food by-products, the total moisture diminution varied from 50 to 75% with a dehydration rate of 4.9 to 12.5% moisture loss per hour of drying [32].

As it was expected, microwave powers had a high impact on the moisture ratio (MR) transformation of the PP samples, which were dried faster than those that underwent convective drying. Thus, the necessary times for CD to have weight stability at different temperatures were 450 ± 10, 303.33 ± 5.77, 166.67 ± 5.77, 160 ± 0, and 96.67 ± 5.75 min for 40, 60, 80, 100, and 120 °C, respectively. However, the time for different microwave powers, i.e., 200, 400, 600, and 800 W, was much lower at 60.0 ± 0.5, 40.0 ± 6.4, 23.17 ± 2.02, and 25.17 ± 4.07 min, respectively. This is consistent with what M’hiri et al. [33] observed when using microwave to dry lemon by-product. Their kinetics indicated a significant decrease in drying time when power rose from 90 to 350 W. Accordingly, the drying time found for 350 W was 3.80 times lower than that found for 90 W. Darvishi et al. [34] explained this phenomenon as a sudden mass transference inside a product when the power heat is greater since it generates more heat in the product, causing a significant steam force disparity between the core and the exterior of the sample owing to the featured microwave’s volumetric hotness.

When comparing the two studied drying processes, it is quite clear that MD needs much shorter periods than CD. Similar results were found in literature regarding CD and MD processes of several vegetables, such as ginger [35], celery leaves [36], coriander leaves [13], and tomato slices [10]. This can be explained by the fact that treatment with microwave has a better effect on the cell membrane permeability of PP, thus enhancing its water diffusivity. In addition, microwave offers an accelerated drying effectiveness with regard to the moisture transmigration versus the medium from a scientific point of view [37].

The loss of moisture rate as a function of time depends also on the nature of the samples, as noticed by Saavedra et al. [38], who found that the drying time was shorter in avocado peels than it was in avocado seeds. Meanwhile, Sozzi et al. [39] demonstrated that moisture ratio diminished constantly with drying time; however, there was no continuous drying rate duration for blackberry by-product. It shows that drying can be considered to occur in a dropping-rate period, where the internal molecular diffusion is the predominant process of mass transfer.

#### 3.1.2. Drying Rate vs. Moisture Content

**Figure 2:**
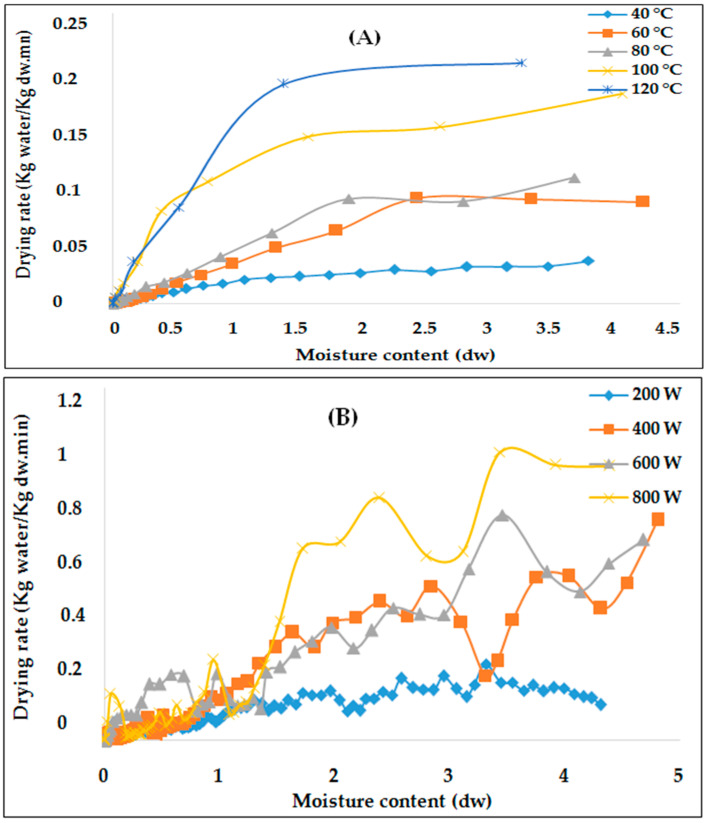
Fluctuations of drying rate versus moisture content using (**A**) convective drying and (**B**) microwave drying. The various temperatures adopted in the convection drying (CD) are 40, 60, 80, 100, and 120 °C, and the powers used in the microwave drying (MD) are 200, 400, 600, and 800 W.

The variation in the drying rate (DR) against the moisture content (Figure 2A,B) reveals that using CD results in an elevated moisture loss at the first step of drying compared to the final stage for all the adopted temperatures. On the contrary, for MD, the different powers used do not follow a homogeneous trend, except for the lowest power (200 W), which is similar to CD. This is probably because of the great water contents of the samples, which activates the diffusion process and generates an elevated DR that decreases with time.

It is emphasized that at the end of the drying process, unexpected data are found (Figure 2B) with huge variations in the drying rate vs. the moisture content (dw). Both decrease and increase in the drying rate can be observed with decreasing moisture content (dw). The possible explanation for these results is that the removal of remaining moisture, which is bound to the matrix, is difficult and does not take place in a homogeneous trend. Other unexplained mechanisms may also take place during this period.

The DR increases with increasing temperatures since the DR averages are 0.01, 0.02, 0.02, 0.05, and 0.05 kg water/kg dw min for 40, 60, 80, 100, and 120 °C, respectively. Accordingly, Ramos et al. [31] demonstrated that smaller temperatures showed less variance among the first and last DRs and exhibited a reduced DR when compared to elevated temperatures for uvaia by-product convection drying. In addition, an elevated moisture loss rate using elevated temperatures was observed for several plant by-products, including lemon wastes [40,41].

The DRs obtained for the microwave drying process are 0.07, 0.11, 0.20, and 0.17 kg water/kg dw min for 200, 400, 600, and 800 W, respectively. It is observed that above 600 W, the DR decreases. According to Drosou et al. [42], in the beginning, the DR is elevated since there is a slight resistivity to heat and mass flux, but when the process advances, the rate diminishes because of the dry sheet that is created surrounding the product, which inhibits heating transfer to the internal face of the product. Furthermore, these results suggest that residues dried at higher powers suffer a solidification incident that precludes the liberation of water, hence diminishing the DR. The solidification could be due to the accumulation of sugars and salts that shift to the external layer alongside water and then harden to develop an impenetrable film that inhibits the transfer of residual water [41].

It is important to note that the best DRs are attributed to MD, which agrees with the data in the literature. The explanation that can be given for this is that microwaves induce fast evaporation of water molecules since they quickly absorb the energy that is responsible for the increase in DR [12].

### 3.2. Kinetic Modeling of Drying

To scale up, plan, and regulate a drying process, mathematical primary and secondary modeling should be viewed as a crucial phase. Additionally, a carefully chosen mathematical equation provides information about the kinetics of the process based on its parameters. The ability to forecast the drying course and assess the process’s conclusion is provided by modeling the drying kinetics [43].

Moreover, a carefully chosen mathematical equation provides information on the kinetics of the process as a function of the constant parameters of the model. These constants play a major role in the validation of the secondary models, which describe the physical phenomenon.

The modeling of the drying kinetics allows us to predict the course of the drying process and to evaluate the conclusion of the process.

The experimental moisture loss was fitted to the empirical mathematical modeling of the drying curves, called the primary model (Table 1). The suitable model was chosen based on the best R^2^ and smallest χ^2^ and RMSE values, and the constants a, b, k, and k_1_ play a role in the validation of the secondary model, which describes the necessary energies of the drying kinetics.

Among the 22 models analyzed in this work, only the Logistic, Sledz et al., and Fernando and Amarasinghe models showed adequate statistical parameter values (the highest R^2^ values and the lowest χ^2^ and RMSE values) (Table 2). Nevertheless, the resulting values of R^2^, χ^2^, and RMSE from the Sledz et al. model revealed that it was the most relevant one to explain the drying procedures of PP using CD and MD. In this model, R^2^ varied from 0.9995 to 0.9999 for CD and from 0.9829 to 0.9997 for MD. The χ^2^ values were null in the case of CD and ranged from 0.0000 to 0.0010 for MD, while RMSE varied from 0.0030 to 0.0054 for CD and from 0.0053 to 0.0304 for MD. Several studies have shown the Sledz et al. model as the most suitable for drying either fruits, vegetables [10,44], or plants [13,36,43]. 

### 3.3. Effective Moisture Diffusivity (D_eff_)

Regarding D_eff_, elevated values were attributed to higher temperatures and powers, usually ranging from 10^−9^ to 10^−11^. In CD, the D_eff_ was 0.20 × 10^−10^ m^2^/s at 40 °C and 1.18 × 10^−10^ at 120 °C, while in MD, it was 0.79 × 10^−10^ m^2^/s at 200 W and 2.09 × 10^−10^ m^2^/s at 800 W. The attained results are in agreement with those reported by several authors who have worked on the drying of other by-products, including uvaia by-product [31], brewers’ spent grain [45], passion fruit peels [46], pomegranate by-products [47] and tomato waste [48].

### 3.4. Energy Consumption and Energy Efficiency (EE)

Drying is one of the actions that consume major energy during food processing. Methods that try to reduce energy expenses are recommended not only for economic reasons, but also to avoid the diffusion of energy to the environment [16]. Thus, an assessment of energy consumption is of major importance when drying is carried out, especially on an industrial scale. In this current study, the energy consumed was determined either for CD or MD after calculating the SECe and EE (Table 3). It was recorded that the SECe values were inversely proportional while those of EE were proportional to the used temperatures and powers. So, the highest SECe values of 245 × 10^7^ MJ/kg and 27.3 × 10^7^ MJ/kg were obtained at 40 °C and 200 W, and the lowest values of 62.7 × 10^7^ MJ/kg and 13.4 × 10^7^ MJ/kg were obtained at 120 °C and 800 W, respectively. The EE values varied from 0.097 × 10^−3^ to 0.380 × 10^−3^% for CD when temperatures varied from 40 to 120 °C, and the values varied from 0.826 × 10^−3^% to 1.68 × 10^−3^% for MD when powers varied from 200 to 800 W. From the results obtained, we can deduce that raising the temperature might reduce the use of energy since the time required for drying is diminished. This is explained by an amelioration of the thermal magnitude and the speeding up of moisture removal [49].The same findings were noticed for MD: energy decreased when the microwave power level was increased. It could be explained by the fact moisture diffusivity is considerably effective when power is elevated, which could be a consequence of cellular modifications that causes fast moisture liberation, thereby diminishing the drying period and reducing energy utilization [50]. 

Based on our observations in this study, we can conclude that CD requires lot of energy compared to MD, which possesses the best EE. Çetin [48] claimed that MD was considered the most effective drying method for tomato waste regarding energy (8.67%) and thermal effectiveness (7.49%). Accordingly, Yi et al. [51] stated that MD is recognized as being twice as efficient as CD for several food products with respect to energy consumption.

### 3.5. Effect of Drying Parameters on Antioxidant Contents 

No studies have previously investigated the effects of different drying temperatures or powers on the antioxidant contents of PP.

#### 3.5.1. Total Phenolic, Total Flavonoid Contents, and Polyphenol Indexes

The total extractable polyphenol content (TPC) of PP (Table 4) was significantly (*p* ≤ 0.05) higher for the samples dried at 40 °C (557.41 mg GAE/100 g dw) and 120 °C (514.94 mg GAE/100 g dw). In the case of MD, the greatest TPC was found in the sample dried at 200 W (767.10 mg EAG/100 g dw), and the lowest TPC was obtained for the sample dried at 600 W (307.90 mg GAE/100 g dw). The highest polyphenol index was attributed to the samples dried at the lowest (40 °C and 200 W) and highest (120 °C, and 400 and 800 W) temperatures and powers. Flavonoids are among the most important classes of phenolic compounds. The same behaviour was observed for TPC, meaning that the highest TFC was attributed to the sample dried at 40 °C (176.71 mg RE/ 100 g dw) and 200W, but the highest temperature (120 °C) and power (800W) also presented a remarkable amount (156.10 mg ER/ 100 g dw and 213.04 mg ER/ 100 g dw, respectively).

Melini et al. [52] stated that during CD of agri-food wastes, a linear TPC decrease occurs with rising temperatures. Valadez-Carmona et al. [53] showed that lower temperatures and prolonged duration of treatment maintain an elevated content of cacao pod husk phenolics. Moreover, TFC diminishes as the drying temperature is augmented [54], as in the case of orange peels [55], in which a high drying temperature causes the degradation of flavonoids. Esparza-Martínez et al. [56] also described a decrease in the content of non-extractable flavonoids of lime as the drying temperature rises. Thus, polyphenols and, among them, flavonoids seem to be degraded after being exposed to the thermal process generated under the effects of both temperature and microwave powers [57]. Moreover, phenolics may be deteriorated by enzymatic oxidation throughout air drying, which leads to cellular degradation favoring the substrate–enzyme link.

M’hiri et al. [33] reported that in a dried product and in the absence of water, compounds are bounded, and, presumably, the extraction with solvents becomes more arduous; hence, the total extraction yield could be minor. Patrón-Vázquez et al. (2019) specifically proposed that phenolics can associate with proteins, or their structure suffers modifications during drying, which could prevent their extraction.

According to Pinto et al. [32], it is essential to keep a low temperature during drying to preserve nutritional value and to avoid losing components since the Maillard reaction speeds up with high temperatures. This is in agreement with the studies by Sozzi et al. [39] with blackberry wastes and Abd Rahman et al. [57] with pomelo peels.

On the other hand, thermal treatment, such as pasteurization of orange pulp by-product, maintained TPC and vitamin C content, although it decreased antioxidant activity and total dietary fibre [58]. Chen et al. [55] indicated that in CD, elevated temperatures of 70 to 100 °C led to an increase in TPC for orange peels, and the highest content of TPC was reached at 100 °C. Spoladore [59] studied the drying of passion fruit by-products and found an increase in TPC from 5.3 to 6.8 mg GAE/g dw for tests that ranged from 60 to 90 °C. Esparza-Martínez et al. [56] showed that a rise of 18.8% in extractable phenolics and 28.3% in flavonoids was obtained when mandarin waste was dried at 120 °C and at the highest power of 800 W, obtaining a considerable content (213 mg Rutin equivalent (RE)/g 100 dw). These increases were probably due to the major accessibility of phenolic precursors because of the non-enzymatic inter-transformation among phenolic substances. Additionally, according to Patrón-Vázquez et al. [41] and Abd Rahman et al. [57], high temperatures promote phenolic extraction probably by improving their solubility and release from cellular wall, where they are associated with macromolecules. Sozzi et al. [39] showed that phenolic decomposition is linked to water availability, so elevated temperatures reduce water activity and, hence, can restrict the decomposition reactions, mainly those produced at intermediate temperatures. M’hiri et al. [33] indicated that low-temperature CD (50 °C) and low microwave power (90 W) are responsible for a reduction of 66% of TPC. It is because of the long-time drying exposure, which may degrade some molecules. Nevertheless, drying at a high temperature (75 °C) and a high microwave power (350 W) for a brief time preserves phenolics better, with a reduction of 52% for MD and 59% for CD.

In summary, the drying impact on phenolic release in by-products is due to micro-structural modifications, which can be positive, neutral, or negative [53]. Several authors have reported a substantial effect on phenolic compounds [33,41,53,57]; however, others have stated that there is no significant change in the concentration of these compounds [31,37]. The observed discrepancies can be attributed to several factors. The drying of pomelo waste revealed that TPC depends on the part of the by-products being dried. Furthermore, the processing treatment, the types of plant matrices, the time duration, and the parameters, including temperature and light strength, could all have an impact on phenolic contents [57].

The comparison of the two drying processes in this current study showed a better retention of phenolics for MD than for CD. Yang et al. (2020) revealed the same trend for various food by-products [37]. As reported by Çetin [48], tomato waste dried in microwave contained the highest flavonoid content. In mango peel and seed and muscadine pomace, MD enhanced the phenolic contents [9]. Cacao pod husk samples dried by microwave demonstrated higher phenolic content compared to the samples dried in an oven [53]. Melini et al. [52] stated that MD produces lower phenolic deterioration in agri-food wastes due to a shorter processing time. In the usage of MD, energy is assimilated instantly and converted into heat, hence rising the temperature of the whole product. Water steam is consequently conducted out of the product by a quick pressure increase, and this releases the bounded phenolic compounds from the matrix cell. In addition, MD could disassociate some phenolics, thereby modifying their structures, transforming insoluble phenolics into more soluble ones, and aiding in driving phenolics to become more accessible for quantification [53].

#### 3.5.2. Chlorophyll and Carotenoid Content

Temperature is one of the most important factors in colour deterioration during by-product drying. In CD, chlorophyll content is influenced by the different temperatures applied, being lowest in samples dried at high temperatures. These results are in agreement with those obtained by several authors [60,61]. Drying of chives at 60 and 65 °C resulted in a reduction of 61.7% of total chlorophyll, 63.0% of chlorophyll a, and 58.1% of chlorophyll b. Chlorophyll degradation is caused by the action of chlorophyllase and lipoxidase, which have higher activity at moderate temperatures than at low ones [60]. In addition, the transformation of chlorophyll to chlorophyllide by chlorophyllase is temperature dependent. It is activated at a moderate temperature, but its effect declines severely when the temperature exceeds 80 °C [61]. Moreover, chlorophylls a and b are degraded to pheophytin a and b, respectively, following first-order kinetics, and chlorophyll a is more heat sensible than chlorophyll b [62].

Regarding MD, low powers are better to maintain chlorophylls, as it has been reported in previous articles [60,62,63]. The great stability of chlorophyll b is remarkable. Roshanak et al. [62] highlighted that green tea chlorophyll *a* was damaged more than chlorophyll b during microwave drying.

In the present study, both processes of drying demonstrated a substantial impact on the content of chlorophylls in the PP samples. However, the microwave drying process allowed the retention of greater amounts of chlorophylls than CD. According to [60], MD showed the best chlorophyll retention since the effect of chlorophyllase and lipoxidase was considerably diminished because of an elevated dehydration rate and shortened drying duration, and moreover, oxygen was limited during microwave-vacuum drying. 

It is assumed that chlorophylls are vulnerable to heat and their preservation is conditioned by temperature and heating time [62]. Consequently, the PP samples dried by CD had the smallest chlorophyll content. Similarly, according to Alibas et al. [63], CD reduced chlorophyll content more than MD.

In the case of carotenoid contents, for the majority of vegetables, drying usually leads to 10–20% of their loss [64]. In our study, the carotenoid content was lower at high temperatures. Carotenoids are highly unsaturated, so they can be oxidized rapidly to form simple molecules, and heat is an important factor that can promote their isomerization [60].

Carotenoid content diminished at high temperatures in tomato peel [65] and seed pumpkin by-products [66], and drying at 50 °C is adequate to stabilize their levels.

The contents of carotenoids were enhanced when the CD temperature ranged from 40 to 60 °C. However, when drying was carried out at 70 °C, the minimum concentration was achieved. Drying at higher temperatures modified the totality of the matrix, thereby weakening plant tissues. Heat can alter cell membranes’ structure and their walls, promoting the delivery of pigments from plant tissues [67]. On the other hand, Song et al. [68] observed that microwave power affected carotenoid concentration considerably and a minor content was found at elevated powers. This can be attributed to the quick increase in the sample temperature, which speeds up the degradation of carotenoids. Moreover, MD decreases lipoxygenases activity, which catalyzes carotenoid decomposition. It is due to the elevated dehydration rate and shortened drying period, and, thus, oxygen accessibility is scarce [60].

The results from this work show that the two drying methods differ in their effects on carotenoids, with MD being more efficient, as it has been previously shown in the literature; for example, in sweet potato, MD retains 80% of carotenoid content compared to 40% for CD [69]. Similarly, the carotenoid contents in carrot pomace were less diminished when using MD (20%) than CD (25%) [70]. It seems that CD is responsible for major carotenoid loss, and it could be because of the fast heating rate and lower oxygen exposure associated with a vacuum microwave [68,69].

### 3.6. Effect of Drying Parameters on Antioxidant Capacity

Antioxidant capacity was assayed based on the scavenging activity on DPPH and phosphomolybdate analyse. DPPH free radical scavenging activity was reduced markedly with increases in drying temperatures and powers, being 14.26 ± 0.06 µM Trolox equivalent/mg dw at 40 °C versus 08.61 ± 0.01 µM Trolox equivalent/mg dw at 120 °C, and 18.20 ± 0.07 µM Trolox equivalent/mg dw at 200 W versus 10.88 ± 0.06 µM Trolox equivalent/mg dw at 800 W (Table 4). Similar results have also been found by other authors using CD or MD to dry food by-products. Indeed, Hernández-Ortega et al. [70] showed that drying carrot pomace in microwave or conventional oven reduced the antioxidant effect, producing a 10% loss in the DPPH assay, when using elevated temperatures. Patrón-Vázquez et al. [41] studied the effect of temperature on the antioxidant activity of lemon wastes, showing that lowest temperatures (40 °C and 50 °C) gave the best values for the DPPH assay.

The drying of blackberry by-products showed that a low temperature yielded a superior antioxidant activity [39]. Furthermore, M’hiri et al. [33] found that microwave drying of lemon by-product at 160 W has the highest radical scavenging activity, but it decreases about 37% at 350 W. Therefore, an increase in microwave power causes a higher temperature, which can lead to the formation of novel constituents (Maillard products and polymerization products) with pro-oxidant effects. Besides, interactions among phenolics, modifications in their structures, or degradation products happen throughout the heating process, which has an impact on antioxidant properties and produces positive and/or negatives synergies on the antioxidant capacity [56]. The diminution in the antioxidant capacity after drying could be related to the diminution in the phenolic and carotenoid contents [70].

In the same way, the samples dried at low temperatures and powers showed the best ability to reduce Mo^+5^ to Mo^+6^ in the phosphomolybdate assay (Table 4).

The comparison between the two drying methods, CD and MD, revealed that the microwave-dried samples showed better antioxidant capacity in both assays. This result is expected since the use of microwaves to dry PP may release antioxidants (polyphenols, carotenoids, and chlorophylls), thereby enhancing the antioxidant capacity when compared to CD. Several authors also reported similar phenomena during the dehydration of other food by-products, such as carrot pomace [70], cacao pod husks [53], and tomato waste [48].

## 4. Conclusions

For potato peel drying, the Sledz model is the most adequate for both tested methods, namely convective (40, 60, 80, 100, and 120 °C) and microwave drying (200, 400, 600, and 800 W). Drying time is reduced with rising temperature and power, needing 27 min at 800 W and 90 min at 120 °C. Antioxidant contents and antioxidant capacity were employed as the quality characteristics of the dried by-product. Both drying methods generate decreases in total phenolics, flavonoids, chlorophylls, and carotenoids, and antioxidant capacity when temperatures or powers increase. The microwave drying method allows the highest retention of different antioxidants and presents the dried samples with the best antioxidant activity. Thereby, microwave drying is the most efficient method regarding time duration in comparison with a convective drying process, and moreover, it is a suitable process for food by-product preservation because of the lower antioxidant content and antioxidant capacity loss. The present study examining the drying of vegetable by-products, such as potato peels, and their effects on bioactive compounds demonstrates a need to study potential preservation processes in order to propose these by-products as novel ingredients to be included in food formulations, following the premises of the circular economy.

## Figures and Tables

**Table 1:** Mathematical models proposed for the drying process [24].

Model Name	Mathematical Equation
Newton	MR = exp(−kt)
Henderson and Pabis	MR = a.exp(−kt)
Logarithmic	MR = a.exp(−kt) + c
Page	MR = exp(−kt^n^)
Modified Page 1	MR = exp(−(kt)^n^)
Modified Page 2	MR = exp(−kt)^n^
Midilli et al.	MR = a.exp(−kt^n^) + bt
Two terms	MR = a.exp(−kt) + b.exp(−k_1_t)
Two-term exponential	MR = a.exp(−kt) + (1 − a).exp(−kat)
Approximation of diffusion	MR = a.exp(−kt) + (1 − a).exp(−kbt)
Verma et al.	MR = a.exp(−k) + (1 − a).exp(−k_1_t)
Modified Henderson and Pabis	MR = a.exp(−kt) + b.exp(−k_1_t) + c.exp(−k_2_t)
Parabolic	MR = a + b.t + c.t^2^
Wang and Singh	MR = 1 + a.t + b.t^2^
Chavez-Mendez et al.	MR = (1−(1−L_2_)L_1_t)^1/(1−L^_2_^)^
Logistic	MR = b/(1 + a.exp(k.t))
Sledz et al.	MR = b.exp(−kt)/(1 + a.exp(k_1_.t))
Simplified Fick’s diffusion equation	MR = a.exp(−k(t/L^2^))
Weibull	MR = exp(−(t/a)^b^)
Demir et al.	MR = a.exp(−kt)^n^ + b
Taghian Dinani et al.	MR = a.exp(−((t − b)/a)^2^)
Fernando and Amarasinghe	MR = (1 + a.t + b.t^2^)/(1 + c.t)

Note: k, k_1_, and k_2_—drying coefficients (1/min); a, b, c, L_1_, and L_2_—coefficients of the equations; n—exponent; t—time (min); L—half of thickness (m).

**Table 2:** The best models’ statistical parameters, drying constants, and coefficients’ values for potato peel drying.

Model	Drying Conditions		Drying Constants and Coefficients				Statistical Parameters		
							R^2^	χ^2^	RMSE
Logistic	CD	40 °C	b = 1.5349	a = 0.5530	k = 0.0202		0.9997	0.0000	0.0050
		60 °C	b = 2.5463	a = 1.5129	k = 0.0347		0.9987	0.0001	0.0089
		80 °C	b = 2.1057	a = 1.1018	k = 0.0447		0.9996	0.0000	0.0051
		100 °C	b = 1.4379	a = 0.4489	k = 0.0650		0.9995	0.0001	0.0068
		120 °C	b = 1.3997	a = 0.3983	k = 0.1190		0.9999	0.0000	0.0038
	MD	200 W	b = 1.9942	a = 0.9524	k = 0.0906		0.9995	0.0000	0.0065
		400 W	b = 54.8324	a = 52.7941	k = 0.1381		0.9949	0.0003	0.0173
		600 W	b = 2.9118	a = 1.9354	k = 0.1956		0.9951	0.0004	0.0196
		800 W	b = 756,811.6567	a = 790,586.2429	k = 0.2044		0.9828	0.0010	0.0304
Sledz et al.	CD	40 °C	b = 3.6874	k = 0.0217	a = 2.7143	k_1_ = −0.0191	0.9997	0.0000	0.0047
		60 °C	b = 1.1901	k = 0.0304	a = 0.1909	k_1_ = −0.1269	0.9995	0.0000	0.0054
		80 °C	b = 1.4528	k = 0.0409	a = 0.4552	k_1_ = −0.0653	0.9997	0.0000	0.0043
		100 °C	b = 11.9400	k = 0.0875	a = 10.9565	k_1_ = −0.0680	0.9999	0.0000	0.0030
		120 °C	b = 1.5087	k = 0.0902	a = 0.5087	k_1_ = −2.6579	0.9998	0.0000	0.0039
	MD	200 W	b = 1.5210	k = 0.0818	a = 0.5130	k_1_ = −0.1405	0.9997	0.0000	0.0053
		400 W	b = 1.0637	k = 0.1419	a = 0.0767	k_1_ = −0.7370	0.9954	0.0003	0.0165
		600 W	b = 1.0101	k = 0.1535	a = 0.0000	k_1_ = 0.8702	0.9990	0.0001	0.0086
		800 W	b = 0.9573	k = 0.2044	a = 0.0000	k_1_ = 0.5734	0.9829	0.0010	0.0304
Fernando and Amarasinghe	CD	40 °C	a = −0.0063	b = 0.0000	c = 0.0049		0.9923	0.0006	0.0241
		60 °C	a = −0.0102	b = 0.0000	c = 0.0190		0.9896	0.0007	0.0255
		80 °C	a = −0.0138	b = 0.0000	c = 0.0193		0.9934	0.0005	0.0219
		100 °C	a = −0.0188	b = 0.0001	c = 0.0150		0.9919	0.0009	0.0266
		120 °C	a = −0.0308	b = 0.0002	c = 0.0314		0.9905	0.0013	0.0305
	MD	200 W	a = −0.0378	b = 0.0004	b = 0.0135		0.9961	0.0003	0.0180
		400 W	a = −0.0542	b = 0.0008	b = 0.0976		0.9908	0.0006	0.0233
		600 W	a = −0.0797	b = 0.0016	b = 0.0704		0.9978	0.0002	0.0130
		800 W	a = −0.0610	b = 0.0011	b = 0.2409		0.9928	0.0004	0.0197

Note: CD—Connective Drying coefficients; MD—Microwave Drying; 40 °C to 120 °C—temperatures used in connective drying; 200 W to 800 W—powers used in microwave drying.

**Table 3:** Results of the time (min) necessary for the stability of weight, DR (kg water/kg dw min), D_eff_ (×10^−10^ m^2^/s), SEC_e_ (×10^7^ MJ/kg H_2_O), and EE (×10^−3^%) calculation for potato peels at different temperatures and microwave powers.

	Convective Drying					Microwave Drying			
	40 °C	60 °C	80 °C	100 °C	120 °C	200 W	400W	600 W	800 W
Time	450 ± 10 ^a^	303.33 ± 5.77 ^b^	166.67 ± 5.77 ^c^	160 ± 0 ^d^	96.67 ± 5.75 ^e^	60.0 ± 0.5 ^a^	40.0 ± 6.4 ^b^	23.17 ± 2.02 ^c^	25.17 ± 4.07 ^d^
DR	0.01 ± 0.00 ^c^	0.02 ± 0.00 ^b^	0.02± 0.00 ^b^	0.05 ± 0.00 ^a^	0.05 ± 0.00 ^a^	0.07 ± 0.00 ^d^	0.11 ± 0.01 ^c^	0.20 ± 0.00 ^b^	0.17 ± 0.01 ^a^
D_eff_	0.20± 0.02 ^e^	0.38 ± 0.02 ^d^	0.53± 0.04 ^c^	0.72± 0.05 ^b^	1.18± 0.05 ^a^	0.79 ± 0.08 ^d^	1.88 ± 0.47 ^b,c^	2.46 ± 0.33 ^a,b^	2.09± 0.23 ^a^
SEC_e_	245 ± 2.46 ^a^	188± 3.90 ^b^	127 ± 3.64 ^c^	127± 4.57 ^c^	62.7 ± 1.98 ^d^	27.30 ± 0.30 ^a^	23.5 ± 0.4 ^b^	12.5 ± 0.1 ^c^	13.4 ± 0.3 ^d^
EE	0.01 ± 0.00 ^d^	0.13± 0.00 ^c^	0.19± 0.01 ^b^	0.19± 0.01 ^b^	0.38± 0.01 ^a^	0.83 ± 0.01 ^d^	0.96± 0.02 ^c^	1.80± 0.02 ^a^	1.68± 0.04 ^b^

Same index letters (^a–e^) show that the mean values are not significantly different at *p* ≤ 0.05 for the same drying method, DR: drying rate; D_eff_: effective moisture diffusivity; SEC_e_: specific energy consumption; EE: Energy Efficiency.

**Table 4:** Results of phenolic, chlorophyll, and carotenoid contents, and antioxidant activity of the extracts of dried PP at different temperatures for convective drying and different powers for microwave drying.

	Convective Drying					Microwave Drying			
	40 °C	60 °C	80 °C	100 °C	120 °C	200 W	400W	600 W	800 W
TPC	557.41 ± 0.75 ^b^	374.26 ± 0.42 ^c^	215.00 ± 2.01 ^e^	180.49 ± 0.15 ^f^	514.94 ± 0.92 ^c^	767.10 ± 1.86 ^a^	337.10 ± 0.15 ^c^	307.90 ± 0.86 ^d^	607.84 ± 0.15 ^b^
PI	6.83 ± 0.06 ^a^	3.68 ± 0.01 ^d^	2.93 ± 0.01 ^e^	1.82 ± 0.01 ^f^	4.53 ± 0.02 ^c^	7.97 ± 0.05 ^a^	5.84 ± 0.03 ^b^	3.38 ± 0.01 ^d^	5.75 ± 0.01 ^b^
TFC	176.71 ± 0.30 ^b^	140.39 ± 0.35 ^c^	129.61 ± 0.99 ^d^	119.41 ± 2.87 ^e^	156.10 ± 0.26 ^c^	222.85 ± 0.32 ^a^	168.86 ± 0.35 ^b^	51.05 ± 0.25 ^c^	213.04 ± 0.21 ^b^
Chl a	1.91 ± 0.02 ^d^	1.58 ± 0.02 ^e^	1.18 ± 0.02 ^f^	0.55 ± 0.01 ^g^	0.42 ± 0.01 ^h^	4.56 ± 0.01 ^a^	3.27 ± 0.03 ^b^	2.56 ± 0.02 ^c^	1.14 ± 0.02 ^f^
Chl b	5.15 ± 0.04 ^d^	3.89 ± 0.03 ^f^	3.10 ± 0.02 ^g^	2.67 ± 0.03 ^h^	2.51 ± 0.04 ^h^	8.72 ± 0.05 ^a^	7.18 ± 0.05 ^b^	6.42 ± 0.04 ^c^	4.39 ± 0.05 ^e^
T Chl	6.34 ± 0.06 ^a^	5.25 ± 0.05 ^b^	4.28 ± 0.04 ^c^	3.22 ± 0.04 ^b^	2.93 ± 0.05 ^d^	13.28 ± 0.06	10.45 ± 0.08	8.98 ± 0.06	5.53 ± 0.07
Caro	0.17± 0.01 ^d^	0.15 ± 0.01 ^e^	0.07 ± 0.00 ^f^	0.06 ± 0.00 ^f^	0.04 ± 0.00 ^g^	1.44 ± 0.01 ^a^	0.86 ± 0.01 ^b^	0.23 ± 0.01 ^c^	0.21 ± 0.01 ^c^
DPPH	14.26 ± 0.06 ^b^	13.23 ± 0.09 ^b^	12.55 ± 0.06 ^c^	10.7 6 ± 0.04 ^e^	08.61 ± 0.01 ^f^	18.20 ± 0.07 ^a^	13.61 ± 2.26 ^c^	11.43 ± 0.05 ^d^	10.88± 0.06 ^e^
TAA	993 ± 60 ^a^	883.2 ± 80.6 ^b^	829.4 ± 61.1 ^b^	596.7 ± 51.2 ^c^	460.8 ± 48.3 ^d^	1006 ± 120 ^a^	924.6 ± 57.4 ^b^	392.2 ± 36.1 ^d^	406.3 ± 35.1 ^e^

TPC: Total extractable polyphenol content (mg GAE/100 g dw); PI: Polyphenol index; TFC: Total flavonoid content (mg GAE/100 g dw); Chl a, b: Chlorophyll a or b (µg/mL); T Chl: Total chlorophylls (µg/mL); Caro: Carotenoids (µg/mL); DPPH: 2,2-diphenyl-1-picrylhydrazyl (µM Trolox equivalent/mg dw); TAA: Total antioxidant activity (µM Trolox equivalent/mg dw). Same letters (^a–h^) in the same row means that the values are not significantly different (*p* ≤ 0.05).

## Data Availability

The data are included in this article. The raw data could be available on request from the corresponding author.
